# Moxibustion alleviates depression-like behavior in rats with Crohn’s disease by inhibiting the kynurenine pathway metabolism in the gut-brain axis

**DOI:** 10.3389/fnins.2022.1019590

**Published:** 2022-12-07

**Authors:** Chunhui Bao, Jin Huang, Huangan Wu, Yueying Ma, Hongyu Zhou, Liming Chen, Dandan Yang, Huirong Liu, Yin Shi, Yuan Lu

**Affiliations:** ^1^Yueyang Hospital of Integrated Traditional Chinese and Western Medicine, Shanghai University of Traditional Chinese Medicine, Shanghai, China; ^2^Key Laboratory of Acupuncture and Immunological Effects, Shanghai University of Traditional Chinese Medicine, Shanghai, China; ^3^Shanghai University of Traditional Chinese Medicine, Shanghai, China; ^4^Hong Kong Baptist University, Hong Kong, Hong Kong SAR, China

**Keywords:** moxibustion, Crohn’s disease, depression, gut-brain axis, tryptophan-kynurenine metabolism

## Abstract

**Background:**

Moxibustion is a potential therapy for inflammatory bowel disease-related depression, but its specific mechanism of action is unclear. This study aimed to investigate the molecular mechanism by which moxibustion alleviates depressive behavior in rats with Crohn’s disease (CD).

**Methods:**

The CD rat model was established with 2,4,6-trinitrobenzenesulfonic acid. Treatment with moxibustion was applied to Tianshu (ST25, bilateral), Qihai (CV6), and Baihui (GV20) acupoints, and the effect of moxibustion was compared with that of the combination of moxibustion plus indoleamine-2,3-dioxygenase 1 (IDO1) inhibitor, 1-methyltryptophan (1-MT). The effects of moxibustion and moxibustion plus 1-MT combination on colonic inflammation and depressive behavior (assessed by forced swimming test, sucrose preference test, and open field test) were investigated. The changes in IDO1, TNF-α, and IL-1β in rat colon and hippocampus were assessed by Western blot (WB). Gas chromatography-mass spectrometry, immunofluorescence staining, and WB were applied to detect kynurenine pathway (KP) metabolites, hippocampal neuronal activity, and microglia activation, respectively.

**Results:**

Both moxibustion and moxibustion plus 1-MT combination significantly alleviated intestinal inflammation and depressive behavior, downregulated the levels of IDO1 in the colon and hippocampus, and inhibited inflammation-inducing factors IL-1β and TNF-α, as well as the kynurenine/tryptophan (KYN/TRP) ratio of KP metabolites, and upregulated the kynurenic acid (KYNA)/KYN ratio and the KYNA/quinolinic acid (QUIN) ratio in the hippocampus in rats with CD; Hippocampal ionized calcium-binding adaptor molecule-1 (Iba-1), c-fos protein expression, activated microglia, and neuronal activation was also significantly reduced by moxibustion and moxibustion plus 1-MT. The addition of 1-MT did not significantly increase the therapeutic effect of moxibustion.

**Conclusion:**

Moxibustion can improve depressive behavior in rats with CD, which may be related to its regulation of KP metabolism in the gut-brain axis and inhibition of hippocampal microglia activation and neuronal activation.

## Introduction

Crohn’s disease (CD), a type of inflammatory bowel disease (IBD), is a chronic inflammatory disease of the gastrointestinal tract characterized by abdominal pain, diarrhea, and weight loss. In recent decades, the prevalence of CD in China has shown an increasing trend (Zhao et al., 2013; Ng et al., 2016). This may be related to several factors including genetic, environmental, dietary, and infection-related factors (Ananthakrishnan, 2015). There is currently no definitive cure for CD (Kaplan, 2015). The disease course is marked by frequent relapses, which greatly affects the life and work of patients. Emotional and psychological factors are known to play an important role in inducing the recurrence of CD (van der Have et al., 2014).

According to two systematic reviews (Mikocka-Walus et al., 2016; Neuendorf et al., 2016), the rate of depression in patients with IBD (21.2%) is almost twofold higher than that in the general population, and the rate of depression in patients with CD may be as high as 24.4%. The prevalence of depression in patients with active CD may even exceed 40%. Many researchers have explained the mechanism of the interaction between IBD and depression through a bidirectional gut-brain interaction (Gracie et al., 2018, 2019). Brain-gut interaction refers to the linkage between the emotional and cognitive centers of the brain with the peripheral control and function of the gastrointestinal tract; it is a bi-directional crosstalk between the central nervous system and the gastrointestinal tract (Wang and Wang, 2016). Tryptophan (TRP) metabolism plays an important role in the gut-brain axis mechanism (O’Mahony et al., 2015). At least 90% of the tryptophan intake in the body is converted to kynurenine for the next step of metabolism, known as the kynurenine pathway (KP) (Badawy, 2017). Clinical studies have shown an association between TRP metabolism and IBD severity (Nikolaus et al., 2017). Dysfunction of key enzymes in the KP was also shown to be associated with IBD and depressive behavior in animal models as well as humans; for example, indoleamine-2,3-dioxygenase 1 (IDO1), a key rate-limiting enzyme in KP metabolism (O’Mahony et al., 2015). Two important metabolites of KP, i.e., kynurenic acid (KYNA) and quinolinic acid (QUIN), have opposite effects on CNS neuronal activity (neurotoxic and neuroprotective properties, respectively) (Chen and Guillemin, 2009). These metabolites can cross the blood-brain barrier to reach the central nervous system; imbalance in the level between KYNA and QUIN has received the most attention in patients with brain dysfunction such as depression (Savitz et al., 2015b; Liu et al., 2018). Specifically, QUIN is mainly metabolized in microglia and has a strong neurotoxic effect on the hippocampus (Lugo-Huitron et al., 2013). Hippocampus is a major component of the brain’s limbic system and is strongly linked to depression (Sheline et al., 2019). Our group demonstrated a close association of increased gray matter volume in the hippocampus and other emotion-related brain areas with depression and anxiety in CD patients (Bao et al., 2015). These findings suggest that the effect of activation of KP on hippocampal function may be one of the key mechanisms of depression in CD.

Treatment of CD with acupuncture and moxibustion has been a popular research area in recent years. Previous work by our team has shown that moxibustion or moxibustion combination with acupuncture can alleviate disease activity (Bao et al., 2014, 2022), and improve serological markers of inflammation (Bao et al., 2014, 2022), immunity (Zhao et al., 2015), and tight junction protein expression in the intestinal wall (Bao et al., 2011; Shang et al., 2015) in CD. In addition, we have also demonstrated that acupuncture can improve depression (Bao et al., 2021) and peripheral KP metabolism (Bao et al., 2016, 2021) in CD, and that the effects of electroacupuncture and moxibustion on brain function in CD patients are related to the brain steady-state afferent processing network and the default mode network, respectively (Bao et al., 2017). Therefore, we speculate that moxibustion may be one of the promising therapies for IBD-related depression; however, the mechanism by which it alleviates depression in CD is still unclear.

This study aimed to investigate the mechanism by which moxibustion alleviates depression in rats with CD. 2,4,6-trinitrobenzenesulfonic acid (TNBS)-induced CD model rats were treated with moxibustion at the Tianshu (ST25, bilateral), Qihai (CV6), and Baihui (GV20) acupoints to investigate whether moxibustion can reduce the depressive behavior by affecting KP metabolism in the gut-brain axis. First, depressive behavioral tests, intestinal inflammation, and IDO1 levels were measured and the correlation between depressive behavior and intestinal inflammation levels was assessed; then, hippocampal KP metabolism inflammation-inducing factor levels, key rate-limiting enzyme IDO1 content, and KP metabolites, hippocampal neuronal activity, and microglia activation were investigated.

## Materials and methods

### Laboratory animals

Clean grade male SD rats, weighing 150 ± 20 g, were obtained from the Animal Experiment Center of Shanghai University of Traditional Chinese Medicine. All rats were raised in SPF-grade feeding rooms in a controlled environment (temperature: 20–25°C, humidity: 40–70%, 12 h light/12 h dark cycle), and acclimatized for 1 week. Animals with normal diet and behavior and without adverse effects were included in the experiment [Experimental Animal Use License: SCXK (Shanghai) 2017-0005]. The experimental protocol complied with the national standard of the People’s Republic of China for ethical review of experimental animal welfare GB/T 358922018 and was approved by the Animal Experimentation Ethics Committee of the Shanghai University of Traditional Chinese Medicine (No. PZSHUTCM200918016).

### Model and methods of interventions

#### Preparation of model

The CD rat models were established according to the academically accepted protocol described by Morris et al. (1989), using 2,4,6-trinitrobenzene sulfonic acid (TNBS, No. P2297, Sigma, USA

Sigma, Novus Biologicals, Invitrogen, Abcam.). Rats were anesthetized by intraperitoneal injection of 2% sodium pentobarbital solution at 0.25 ml/100 g on days 1, 8, 15, and 22, respectively, after 24 h of fasting. Afterward, a mixture of 5% TNBS and 50% ethanol (2:1 ratio) was administered as an enema at a dose of 0.3 ml/100g. After the enema, the rats were held upside-down suspended by lower limbs for 3 min under anesthesia. Rats in the control group were anesthetized by the same method and administered an equal amount of saline by enema.

**FIGURE 1:**
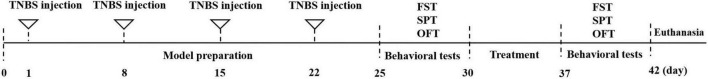
Experimental procedure.

After the completion of modeling, three rats in the modeling and control groups were randomly selected and tested for depression-related behavior. Rats were sacrificed by neck dislocation under anesthesia, and the colon was harvested for hematoxylin-eosin (HE) staining and histopathologic scoring to evaluate whether the model establishment was successful. A schematic illustration of the experimental procedure is presented in Figure 1.

#### Grouping and intervention

##### Experiment I

Rats were randomly divided into 4 groups (*n* = 8/group) after 7 days of acclimatization, and therapeutic interventions were performed after modeling. Rats in the moxibustion group were treated with moxibustion at ST25, CV6, and GV20. These acupoints were selected with reference to previous studies (Bao et al., 2011; Zhao et al., 2019). The moxa strips were made of refined moxa velvet with a diameter of 0.5 cm (Hanyi, Henan Nanyang Han Medicine Moxa Co. Ltd.), ignited, and then applied at 2–3 cm vertically above the acupoints of the rats. The order of moxibustion was ST25 and CV6 first, followed by GV20. The treatment frequency was once every 10 min, once a day, for a total of 7 times (Bao et al., 2019). In the sham group, the moxa strips were not lit and the rest of the operation was the same as in the moxibustion group. Rats in the control group and the model group received no treatment and were only fastened in the same way as rats in the moxibustion group.

##### Experiment II

Rats were randomly divided into 5 groups (*n* = 8/group) after 7 days of acclimatization to the environment. The treatment intervention was performed for 7 days at the end of modeling, once a day, and the modalities of moxibustion were the same as in Experiment 1. In the 1-MT + moxibustion group, the IDO1 inhibitor 1-MT (No. 860646, Sigma, USA) was prepared as 10% 1-MT solution and 1 ml of the solution was injected intraperitoneally into rats with CD half an hour before each moxibustion, and 1 ml of 0.9% saline was injected intraperitoneally into the moxibustion group every day before moxibustion as the control. Rats in the 1-MT group were administered intraperitoneal injection of 1 ml per day, and rats in the control and model groups were administered intraperitoneal injection of 1 ml of 0.9% saline per day.

### Specimen collection and processing

At the end of the intervention, rats in each group were weighed and then fasted for 24 h (with free access to water). Sample collection was performed on the following day. Rats were anesthetized with 2% sodium pentobarbital solution (0.25 ml/100 g intraperitoneally) according to their body mass. Three rats in each group were randomly selected for brain perfusion; hippocampal tissues were collected and soaked in 4% paraformaldehyde and fixed in a refrigerator at 4°C for 24 h for immunofluorescence staining and Western blot assay. After the remaining rats were anesthetized, a 2-cm section of the colon was laid flat on filter paper, and fixed by 4% paraformaldehyde immersion for colon bulk scoring, HE staining, and Western blot assay.

### Assessment of body weight and intestinal inflammation

#### Body weight

The increase in body weight at the end of the intervention in each group was compared with that at baseline.

#### Disease activity index

Rats were evaluated in terms of percentage loss of body mass, fecal traits, and blood in stool, referring to the method of calculating disease activity described by Murthy et al. (1993).

#### Macroscopic damage scoring of tissues

The morphology of colon was examined in each group, and then dissected along the longitudinal axis of the mesentery and washed with PBS solution to examine the gross morphology of the colonic mucosa. The Colonic Mucosal Damage Index (CMDI) was observed visually by referring to Paiotti et al. (2012), including vascular congestion and edema on the surface of the intestinal wall, ulceration, thickness of the intestinal wall, and adhesions.

#### Colonic histopathologic score

Colonic tissues from each group were subjected to HE staining and histopathological changes such as colonic mucosal epithelium, intestinal glands, inflammatory cell infiltration, and granulation tissue proliferation were observed under light microscopy. The grading was performed according to the method developed by Appleyard and Wallace (1995).

### Depressive behavior tests

#### Forced swimming test

The forced swimming experiment was adapted from Petit-Demouliere et al. (2005). Rats were placed in transparent cylindrical (50 cm high, 30 cm diameter) buckets filled with water at a temperature of (23 ± 1)°C and a depth of 30–33 cm, with one rat in each bucket. Rats in the water were neither able to touch the bottom with the hind paws to support the body nor able to climb the barrel wall with the front paws. The cumulative resting time of the rats in the water was recorded, which was the time when the rats floated motionless on the water surface or kept their heads out of the water with only slight movement of the limbs to maintain the body balance. The behavioral performance of the rats was recorded for 6 min and the cumulative immobility time of the rats at the water surface during the last 5 min was analyzed. Each rat was tested individually.

#### Sucrose preference test

The sucrose preference test was adapted from Ko et al. (2020). All animals were trained to adapt and learn to drink sugar water for 48 h before the start of the test. One bottle of 1% sucrose water and one bottle of pure water for experimental animals were placed in each cage for the first 24 h, and the positions of the two bottles were exchanged around the second 24 h. At the end of adaptation period, all rats were taken off water for 4 h and then the sucrose preference test was performed. All rats were kept in a single cage during the experiment and provided two pre-weighed bottles of water, one bottle of 1% sucrose water and another bottle of pure water for experimental animals. The position of the two bottles was changed after 1 h; after 2 h, the two bottles were taken away and weighed. Sucrose preference ratio = total sucrose consumption/[total pure water consumption (g) + total sucrose consumption (g)] × 100%.

#### Open field test

The total path of the open field test was calculated as described by Walsh and Cummins (1976). Three days before the start of the experiment, the rats were stroked in the experimental environment for 5 min every day. During the test, each rat was placed at the center of a 100 × 100 × 50 cm open field box, and the movements of the rats were tracked using the Digbeth animal behavior video tracking analysis system for 5 min. Then the rats were removed, the bottom of the box and the four walls were cleaned, and the residual odor of the experiment was removed with 35% ethanol. A quiet environment was maintained during the test, and the rats in each group were tested alternately. After the completion of the test, the total path traveled for each rat’s activity was analyzed.

### Hematoxylin-eosin staining

Colonic specimens from rats were fixed in 10% formalin for 48 h and dehydrated by passage through a graded ethanol series. The specimens were paraffin-embedded, sectioned, and dewaxed by immersion in xylene. The dewaxed slices were dehydrated by passage through different concentrations of ethanol. The slices were placed in an aqueous hematoxylin solution and eosin staining solution. The slides were placed in xylene for 3 min × 2 times transparent, sealed with neutral gum, then placed in an oven at 65°C for 15 min. Photographs were obtained through a microscope (NIKON Corporation, ECLIPSE Ci) and histopathologic scoring was performed.

### Western blot

Cell lysates were separated by 10% sodium dodecyl sulfate-polyacrylamide gel electrophoresis, transferred to nitrocellulose membranes, and incubated with primary anti-Iba1 antibody (#17198, CST), c-fos antibody (NBP2-50037, Novus Biologicals), IDO1 antibody (PA5-107329, Invitrogen), GAPDH antibody (#5174, CST) for 2 h, followed by incubation with secondary anti-Goat anti-mouse IgG (1:1000, A0216, Beyotime) and Goat anti-rabbit IgG (1:1000, A0208, Beyotime) for 1 h at 37°C. The ECL luminescent solution was added to the front of the membrane for 5 min in the darkroom to visualize the protein, which was placed in an imaging system (Tanon, Tanon-5200) for scanning to detect the reaction bands.

### Immunofluorescent staining

Hippocampus specimens from rats were rinsed with saline at 4°C, fixed in 10% formalin for 24 h, paraffin embedded, and sliced. The slices were dewaxed in xylene, soaked in graded alcohol series for 5 min, and rinsed in tap water for 10 min, respectively. 0.01 M sodium citrate buffer solution was used for high-pressure repair for 15 min, and drops of primary anti-Iba-1 antibody (1:100, ab283319, Abcam), c-fos antibody (1:100, ab214672, Abcam) were added to the wet box and incubated overnight at 4°C. The slices were rinsed thrice with PBS for 3 min each and incubated with the corresponding secondary antibodies donkey anti-mouse IgG (1:500, A0460, Biyuntian) and goat anti-rabbit IgG (1:500, A0423, Biyuntian). DAPI (1:5000 dilution) was added to the anti-quench sealer, sealed, stored in the refrigerator at −20°C, and photographed by fluorescence microscopy (NIKON, ECLIPSE Ni).

### Statistical analysis

Continuous variables exhibiting homogenous variance and normal distribution were presented as mean ± standard deviation and between-group differences were assessed using one-way ANOVA, with *post-hoc* LSD test used for multiple comparisons; paired test was used for within-group comparisons. Continuous variables with non-normal distribution or inhomogenous variance were presented as median (interquartile range), and between-group differences were assessed using the Kruskal–Wallis *H* test; the Nemenyi test was used for multiple comparisons, while the Wilcoxon rank sum test was used for within-group comparisons. The correlation between intestinal inflammation and depressive behavior in each group was analyzed using a typical correlation. Experimental images were analyzed using ImageJ (NIH, USA) as well as Analyze Skeleton (2D/3D) and FracLac plug-in packages. Statistical analyses were performed using SPSS 24.0 (IBM, USA). *P-*values < 0.05 were considered indicative of statistical significance.

## Results

### Experiment I

#### Assessment of body weight and intestinal inflammation

**FIGURE 2:**
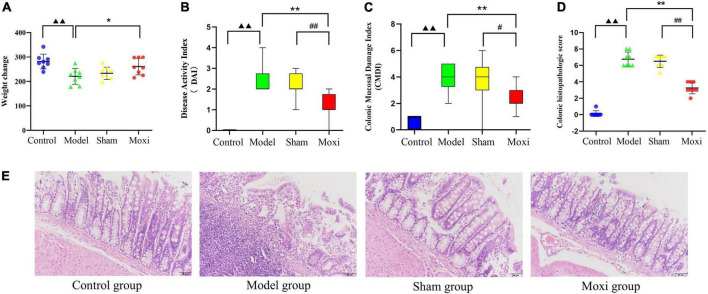
Effect of moxibustion on weight gain and improvement of intestinal inflammation levels. **(A)** Change in body weight, **(B)** Disease activity index (DAI) score, **(C)** Colonic Mucosal Damage Index (CMDI) score, and **(D)** Colonic histopathologic score. **(E)** Hematoxylin-eosin (HE)-stained sections of colon tissue of rats in each group. *n* = 8 per group. ^▲▲^*P* < 0.01 vs. control group; *P < 0.05, **P < 0.01 vs. model group; ^#^*P* < 0.05, ^##^*P* < 0.01 vs. sham group.

The increase in body weight of rats in the model group was significantly lower than that in the control group (*P* < 0.01). A significant increase in weight gain was observed after moxibustion treatment (*P* < 0.05) (Figure 2A). The disease activity index (DAI) score, CMDI score, and colonic histopathologic score in the model group were significantly higher than those in the control group (*P* < 0.01 for all), but all were reversed after moxibustion treatment (*P* < 0.01 for all), and significantly superior to those in the sham group (*P* < 0.01, *P* < 0.05, *P* < 0.01, respectively) (Figures 2B–E). This suggests that moxibustion significantly reduced abnormally high DAI score, CMDI score, and colonic histopathologic score, as well as accelerated weight gain in depressed rats with CD.

#### Behavioral evaluation of depression in rats

**FIGURE 3:**
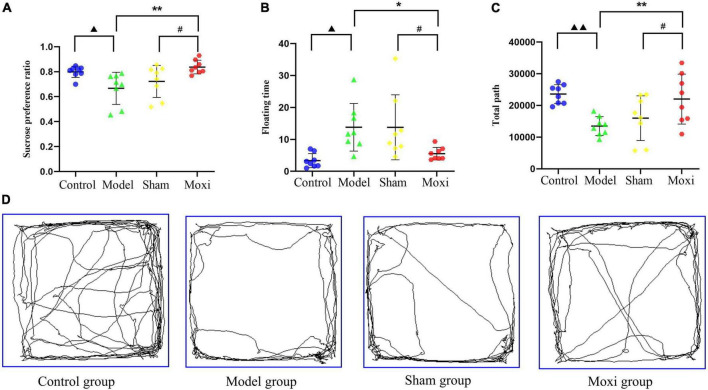
Effect of moxibustion on alleviating depression-like behavior. **(A)** Sucrose preference ratio, **(B)** Floating time, **(C)** Total path, and **(D)** Activity trajectory in the open field experiment of rats in each group. *n* = 8 per group. ^▲^*P* < 0.05 vs. control group, ^▲▲^*P* < 0.01 vs. control group; *P < 0.05, **P < 0.01 vs. model group; ^#^*P* < 0.05 vs. sham group.

To evaluate whether the modeling was successful and to assess the therapeutic effect of moxibustion, we performed behavioral tests on rats. In the sucrose preference test (SPT), rats in the model group had a significantly lower sucrose preference ratio compared with the control group (*P* < 0.05), and rats in the moxibustion group had a significantly higher sucrose preference ratio after treatment (*P* < 0.01), which was significantly better than that in the sham group (*P* < 0.05) (Figure 3A). Moxibustion significantly reduced the floating time of rats (*P* < 0.05) which was significantly better than that in the sham group (*P* < 0.05) (Figure 3B). In terms of open field test (OFT), the total path in the model group was significantly reduced compared with the control group (*P* < 0.01), and the total path was significantly increased after applying moxibustion (*P* < 0.01) and significantly better than that in the sham group (*P* < 0.05) (Figures 3C, D). The results suggest that moxibustion can effectively alleviate the depressive state of rats with CD.

#### Correlation analysis of intestinal inflammation and depressive behavior in each group

The sucrose preference ratio of rats also showed a significant positive correlation with body weight gain (*r* = 0.514, *P* < 0.01). The sucrose preference ratio and total path showed a significant negative correlation with DAI score, CMDI score, and histopathologic score. The floating time of rats showed a significant positive correlation with DAI score (*r* = 0.583, *P* < 0.001) and histopathologic score (*r* = 0.42, *P* < 0.05).

#### Protein expression levels of IDO1, IL-1β, and TNF-α in the colon

**FIGURE 4:**
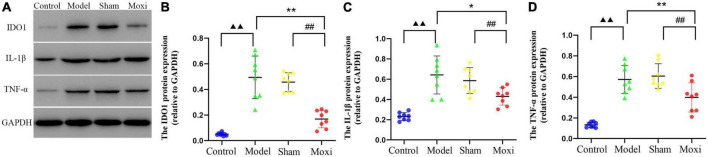
Effect of moxibustion on protein expressions of IDO1, IL-1β, and TNF-α in the colon. **(A)** Western blot bands of IDO1, IL-1β, and TNF-α protein expression, **(B)** IDO1 protein, **(C)** IL-1β protein, and **(D)** TNF-α protein expression in the colon of rats in each group. *n* = 8 per group. ^▲▲^*P* < 0.01 vs. control group; *P < 0.05, **P < 0.01 vs. model group; ^##^*P* < 0.01 vs. moxibustion group.

Colon IDO1 protein expression in the model group was significantly greater than that in the control group (*P* < 0.01), suggesting high expression of IDO1, a key rate-limiting enzyme for KP metabolism, and accelerated KP metabolism. Compared with the model group, the expression of IDO1 protein was significantly decreased in the moxibustion group (*P* < 0.01), and the expression was lower than that in the sham group (*P* < 0.01) (Figures 4A, B). The expression of colonic IL-1β protein and TNF-α protein was significantly higher in the model group compared to the control group (*P* < 0.01 for both), suggesting that the increased expression level of IDO1-inducing factor (IDO1 is a rate-limiting enzyme of intestinal KP metabolism) is a potential cause of IDO1 activation. Compared with the model group, the expression of both proteins was significantly reduced after moxibustion treatment (*P* < 0.01 for both), and the expression was lower compared to that in the sham group (*P* < 0.01, both) (Figures 4A, C, D). These findings suggested that moxibustion significantly inhibited intestinal inflammation in depressed rats with CD, reduced the high expressions of IDO1-inducing factors IL-1β protein and TNF-α protein, and inhibited the activation of IDO1 protein, which may slow down the metabolism of KP.

### Experiment I

#### Assessment of body weight and intestinal inflammation

**FIGURE 5:**
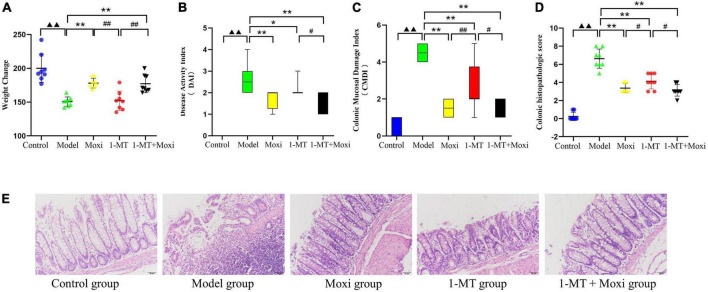
Effects of moxibustion, 1-MT, or a combination of both on weight gain and improvement of intestinal inflammation levels. **(A)** Change in body weight, **(B)** Disease activity index (DAI) score, **(C)** Colonic Mucosal Damage Index (CMDI) score, and **(D)** Colonic histopathologic score. **(E)** Hematoxylin-eosin (HE)-stained sections of colon tissue of rats in each group. *n* = 8 per group. ^▲▲^*P* < 0.01 vs. control group; *P < 0.05, **P < 0.01 vs. model group; ^#^*P* < 0.05, ^##^*P* < 0.01 vs. 1-MT group.

The body weight of rats in the model group was significantly lower than that in the control group (*P* < 0.01), while the DAI score, CMDI score, and colonic histopathologic score were significantly higher than those in the control group (*P* < 0.01 for *all*). After the intervention, DAI score (Figure 5B), CMDI score (Figure 5C), and colonic histopathologic score in the moxibustion group, 1-MT group, and 1-MT + moxibustion group were significantly lower than those in the model group (Figures 5D, E) (*P* < 0.01 for all except DAI score in the 1-MT group, *P* < 0.05); the body weight of rats in the moxibustion group and 1-MT + moxibustion group showed a significant increase (*P* < 0.01) (Figure 5A). The differences between the three groups were not significant. These findings suggested that moxibustion, 1-MT, or the combination of both significantly reduced the abnormally high DAI score, CMDI score, and colonic histopathologic score in depressed rats with CD. Moxibustion and moxibustion combined with 1-MT also contributed to the increase in body mass, but the addition of 1-MT to moxibustion did not significantly increase the effect.

#### Behavioral evaluation of depression in rats

**FIGURE 6:**
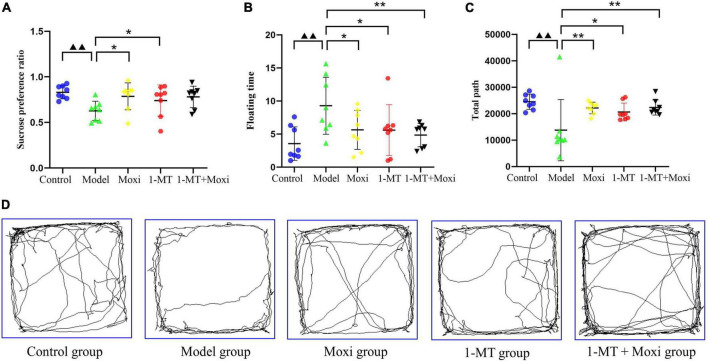
Effects of moxibustion, 1-MT, or a combination of both on alleviating depression-like behavior. **(A)** Sucrose preference ratio, **(B)** Floating time of rats in each group; **(C)** Total path, and **(D)** Activity trajectory of rats in the open field experiment of rats in each group. *n* = 8 per group. ^▲▲^*P* < 0.01 vs. control group; *P < 0.05, **P < 0.01 vs. model group.

The sucrose preference index and the total path of the open field test in the model group were significantly lower than those in the control group (*P* < 0.01 for both), while the floating time was significantly higher (*P* < 0.01), suggesting a depressive state in the rat model of CD. After the intervention, the sucrose preference index in the moxibustion group and the 1-MT group was significantly higher than that in the model group (*P* < 0.05 for both) (Figure 6A); the floating time was significantly decreased in the moxibustion group, the 1-MT group, and the 1-MT + moxibustion group (*P* < 0.05, *P* < 0.05, *P* < 0.01, respectively) (Figure 6B), and the total path of the open-field test was significantly increased (*P* < 0.01, *P* < 0.05, *P* < 0.01, respectively) (Figures 6C, D). However, there was no significant difference between moxibustion group, 1-MT group, and 1-MT + moxibustion group in this respect. These findings suggested that moxibustion, 1-MT, and 1-MT + moxibustion all significantly alleviated depressive behavior in rats with CD, and that the addition of 1-MT to moxibustion did not significantly increase the effect.

#### Morphology of hippocampal neuronal cell activity and microglia

**FIGURE 7:**
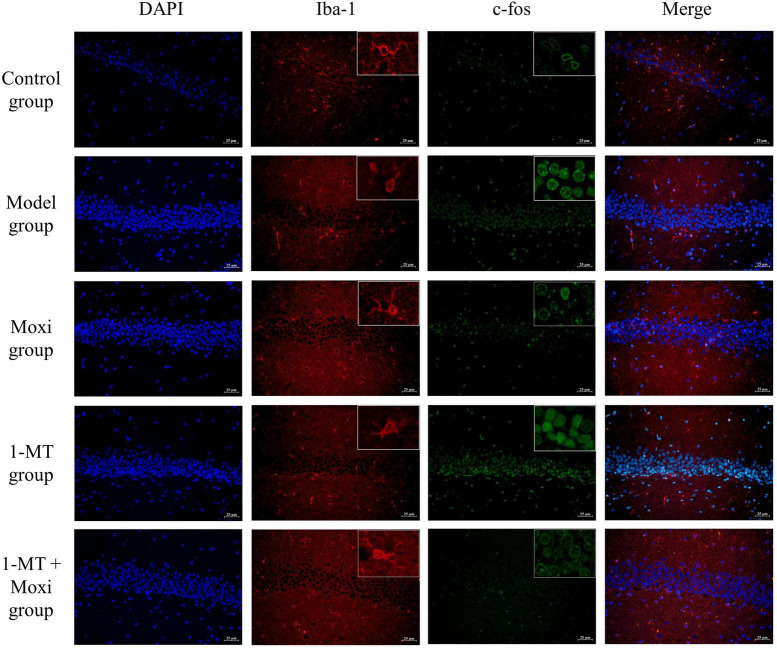
Effects of moxibustion, 1-MT, or a combination of both on improving hippocampal neuronal activity and microglia activation morphology in each group of rats. Representative images of immunofluorescence staining of hippocampal slices for DAPI (blue, nuclei indicator), Iba-1 (red), TLR4 (green), Iba-1 (red) + TLR4 (green). *n* = 3 per group.

The Iba1 microglia in the resting state was branched, with yellow or brownish-yellow cells, mainly distributed in the cytoplasm and intercellular stroma. Compared with the control group, Iba1 expression was increased in the cerebral cortex of the model group rats, and the cell protrusions were retracted with an amoeboid appearance, indicating successful induction of microglia activation; a large number of c-fos neurons were also seen expressed as oval and round brown neurons. After 1-MT, moxibustion, and 1-MT + moxibustion interventions, microglia activation in the hippocampus was reduced in the three groups, and neuronal activation was also reduced, which was improved compared with the model group (Figure 7).

#### Detection of biomarkers in hippocampal neuronal cell and microglia

**FIGURE 8:**
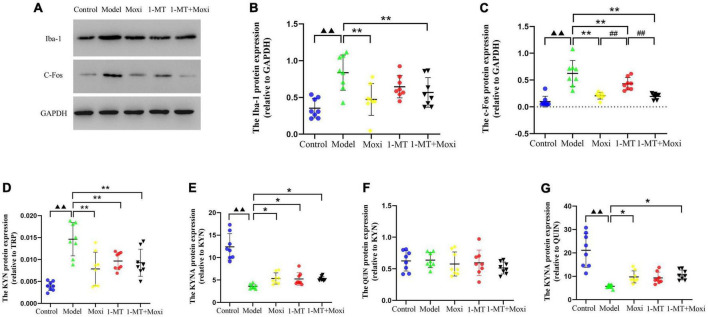
Effects of moxibustion, 1-MT, or a combination of both on improving hippocampal neuronal activity and microglia activation markers in each group of rats. **(A)** Western blot bands of Iba-1 and c-fos protein expression, **(B)** Iba-1 protein expression, **(C)** c-fos protein expression, **(D)** KYN/TRP ratio, **(E)** KYNA/KYN ratio, **(F)** QUIN/KYN ratio, and **(G)** KYNA/QUIN ratio in the hippocampus of rats in each group. *n* = 8 per group. ^▲▲^*P* < 0.01 vs. control group; *P < 0.05, **P < 0.01 vs. model group; ^##^*P* < 0.01 vs. 1-MT group.

Hippocampal c-fos protein and Iba-1 protein expressions in the model group were significantly higher than those in the control group (*P* < 0.01 for both), suggesting abnormal increase in neuronal activity and microglia activation in the hippocampus of depressed rats with CD. After the intervention, hippocampal Iba-1 protein expression (*P* < 0.01 for both) and c-fos protein (*P* < 0.01 for both) were significantly decreased in the moxibustion group and the 1-MT + moxibustion group compared with the model group, while there was no significant change in the Iba-1 protein expression. There was no significant difference between the moxibustion group and the 1-MT + moxibustion group with respect to hippocampal c-fos protein or Iba-1 protein expression (Figures 8A–C). These findings suggested that moxibustion, 1-MT, and 1-MT + moxibustion all significantly reduced the abnormally high expression of hippocampal c-fos and Iba-1 protein in the depressed rats with CD and inhibited the abnormal activation of neurons and microglia, while the combination of moxibustion and 1-MT did not significantly enhance the effect.

#### Detection of hippocampal kynurenine pathway

The hippocampal KYN/TRP ratio in the model group was significantly higher than that in the control group (*P* < 0.01), and the KYNA/KYN ratio and KYNA/QUIN ratio were significantly lower than that in the control group (*P* < 0.01, both), suggesting that the balance between KYNA and QUIN was disrupted by increased hippocampal KP metabolism in depressed rats with CD. After the intervention, compared with the model group, the expression of hippocampal KYN/TRP ratio was significantly decreased (*P* < 0.01 for *all*), and the KYNA/KYN ratio was significantly increased (*P* < 0.05 for *all*) in the moxibustion group, 1-MT group, and 1-MT + moxibustion group. In addition, the KYNA/QUIN ratio was also significantly increased (*P* < 0.05, both) in the moxibustion group and 1-MT + moxibustion group; however, the differences between these two groups were not statistically significant. There was no significant difference between the model group and the control group, or between the hippocampal QUIN/KYN ratios after the intervention (Figures 8D–G). The results indicated that there was increased hippocampal KP metabolism, an imbalance between KYNA and QUIN metabolism, and an increased KYNA/QUIN ratio in the depressed rats with CD compared to the control group. Both moxibustion and 1-MT + moxibustion significantly decreased the hippocampal KYN/TRP ratio and increased KYNA/KYN and KYNA/QUIN ratios in CD depressed rats, suggesting a slowing down of that KP metabolism after the intervention; in addition, the combination of moxibustion and 1-MT did not increase the effect.

#### Protein expression of IDO1—The key rate-limiting enzyme of kynurenine pathway metabolism

**FIGURE 9:**
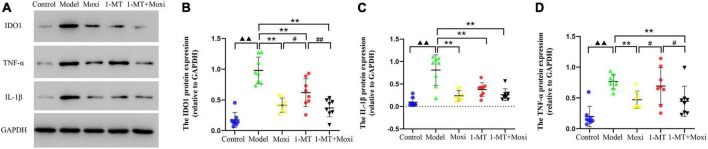
Effects of moxibustion, 1-MT, or a combination of both on protein expression of IDO1, IL-1β, and TNF-α in the hippocampus. **(A)** Western blot bands of IDO1, IL-1β, and TNF-α protein expression, **(B)** IDO1 protein expression, **(C)** TNF-α protein expression, and **(D)** IL-1β protein expression in the hippocampus of rats in each group. *n* = 8 per group. ^▲▲^*P* < 0.01 vs. control group; **P < 0.01 vs. model group; ^ #^*P* < 0.05, ^##^*P* < 0.01 vs. 1-MT group.

Hippocampal IDO1 protein expression in the model group was significantly higher than that in the control group (*P* < 0.01). After the intervention, hippocampal IDO1 protein expression in the 1-MT group, moxibustion group, and 1-MT + moxibustion group were significantly decreased compared with the model group (*P* < 0.01 for all). Hippocampal IDO1 protein expression in the moxibustion group and 1-MT + moxibustion group was lower than that in the 1-MT group (*P* < 0.05, *P* < 0.01, respectively). However, there was no significant difference between the 1-MT + moxibustion and moxibustion groups in this respect (Figure 9). This suggested that moxibustion, 1-MT, and 1-MT + moxibustion all significantly reduced the abnormally high expression of IDO1 protein in the hippocampus of the depressed rats with CD. Moxibustion and 1-MT + moxibustion were more effective than 1-MT, but the combination of moxibustion and 1-MT did not significantly increase the effect of moxibustion.

#### Content of kynurenine pathway’s key rate-limiting enzyme IDO1 regulatory factor—TNF-α and IL-1β

The protein expression of TNF-α and IL-1β in the hippocampus of rats in the model group was significantly higher than that in the control group (*P* < 0.01, both). After the intervention, the hippocampal TNF-α protein expression significantly decreased in the moxibustion group and the 1-MT + moxibustion group compared with the model group (*P* < 0.01, both), and IL-1β protein also significantly decreased, as well as IL-1β protein significantly decreased in the 1-MT group. Compared with the 1-MT group, the hippocampal TNF-α protein expression was lower in the moxibustion and moxibustion + 1-MT groups (*P* < 0.05 for both), while there was no significant difference between the three groups. This suggested that moxibustion and 1-MT + moxibustion significantly reduced hippocampal TNF-α protein in depressed rats with CD, and the effect of the combination was better than that of 1-MT alone. Moxibustion, 1-MT, and 1-MT + moxibustion all significantly reduced the abnormally high expression of IL-1β protein in the hippocampus of depressed rats with CD. The use of 1-MT did not significantly increase the inhibitory effect of moxibustion on TNF-α protein and IL-1β protein.

## Discussion

**FIGURE 10:**
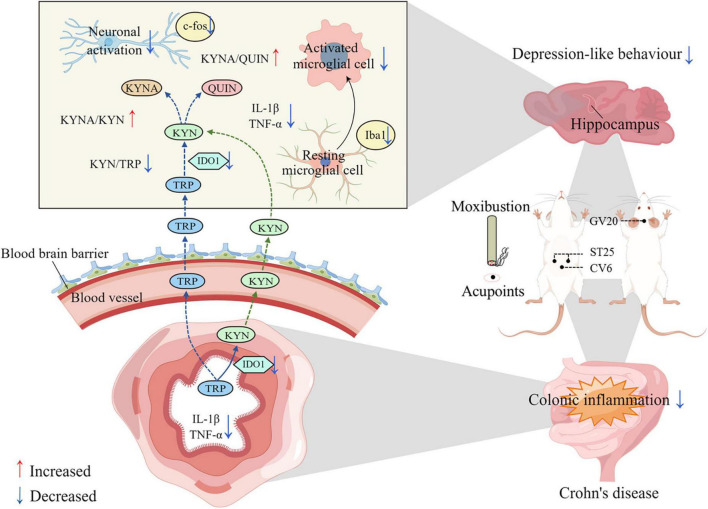
Schematic illustration of this research: the potential mechanism by which moxibustion inhibits gut-brain axis kynurenine pathway (KP) metabolism in the treatment of 2,4,6-trinitrobenzenesulfonic acid (TNBS)-induced depressive behavior in rats with Crohn’s disease (CD). The mechanism of action of moxibustion at ST25, CV6, and GV20 to improve depression-like behavior and intestinal inflammation in rats with CD may be due to the reduction of intestinal inflammatory cytokines, such as IL-1β and TNF-α, after moxibustion treatment. These inflammatory factors further reduced the activation of intestinal IDO1 and inhibited the excessive degradation of TRP to KYN in the intestine. KYN crosses the blood-brain barrier through the gut-brain axis and enters the brain, where it is further metabolized to KYNA and QUIN. In contrast, the increased ratio of KYNA/KYN and KYNA/QUIN, reduced microglia activation and neuronal activity in hippocampus after moxibustion intervention may be one of the mechanisms by which moxibustion alleviates depression-like behavior in rats with CD.

In this study, rats with CD exhibited some behavioral changes akin to those observed in depression. The potential mechanism may involve the gut-brain axis in hippocampal KP metabolism accompanied by hippocampal neuronal activation and increased microglia activity. In contrast, the excessive metabolism of tryptophan by the KP pathway caused by activation of intestinal IDO1 may be one of the important factors contributing to the increase in hippocampal KP metabolites. Our findings suggest that the potential mechanism of action of moxibustion and the combination of moxibustion with 1-MT is through inhibition of the IDO1 and the KP metabolic pathway that mediates the “crosstalk” between the gut and the brain. The depressive behaviors of the restrained rat were greatly reversed by moxibustion or moxibustion + 1-MT. Moreover, the combination of moxibustion and 1-MT was found to be less effective than moxibustion alone. A schematic illustration of the mechanism by which moxibustion alleviates TNBS-induced depressive behavior in rats with CD is shown in Figure 10.

Compared with the control group, the body weight, sucrose preference index, and total path of the open field test were significantly reduced in the model group, but significantly increased after moxibustion treatment; while the DAI score, CMDI score, colonic histopathologic score, and floating time were significantly increased in the model group rats, and significantly reduced after moxibustion treatment. As in rather, TNBS used in the modeling process can cause serious damage to intestinal function affecting both digestion and absorption, and induce depressive manifestations. Intestinal inflammation showed a significant correlation with depression, suggesting that the two develop in parallel. The more severe the intestinal inflammation, the more pronounced was the depression-like behavior. This supports further research into the potential mechanisms between intestinal inflammation and depressive behaviors. On the other hand, IDO1 protein, a key rate-limiting enzyme of KP, has been extensively studied in the control of mood and behavior as well as psychopathogenesis. IDO1 has been shown to be activated by inflammatory cytokines such as IFN-γ, TNF-α, and IL-1β. Studies have found reduced circulating levels of TRP in cancer patients treated with IL-2 or IFN-α (Capuron et al., 2002). Some studies have also found elevated inflammatory cytokines in some depressed patients (Maes, 1995). In the study by Kim et al. (2012) and Lawson et al. (2013), IDO1 pharmacological inhibition or knockdown was found to alleviate depressive behavior in rats. Interestingly, in this study, the expressions of IDO1, IL-1β, and TNF-α proteins of the model group were significantly higher than that of the control, but they became significantly lower after moxibustion or moxibustion + 1-MT, although the addition of 1-MT did not result in a significant enhancement of the effect of moxibustion. This suggested that the therapeutic effect of moxibustion may be mediated *via* decrease in IDO1 protein and its inducing factors IL-1β protein and TNF-α protein, thus reducing intestinal inflammation and depressive behavior by inhibiting KP. Both moxibustion and 1-MT may have a similar mechanism of action; therefore, the addition of 1-MT to moxibustion failed to increase the effect. TRP is one of the eight essential amino acids in the human body. TRP content in the diet is degraded through three main pathways after entering the human body, with the vast majority of TRP ingested into the human body being converted through the IDO1-mediated KP pathway (Agus et al., 2018). IDO1 plays an essential role in KP. Therefore, we hypothesized that the anti-depression effects of moxibustion are mainly mediated through KP and have synergistic effects of multiple metabolites.

After TRP is absorbed through the intestine, part of it is metabolized into subsequent products in the intestine, liver, and other organs, and part of it directly enters the blood circulation (Gracie et al., 2019). TRP, KYN, and some of the products can penetrate the blood-brain barrier (Cervenka et al., 2017). In the intracerebral environment, IDO1 is expressed mainly on microglia and astrocytes. Approximately 40% of the KYN content in the brain is synthesized in the brain and the rest is derived from plasma (Gal and Sherman, 1980). As expected, hippocampal neuronal activity, microglia activation, c-fos protein, Iba-1 protein, and IDO1 protein were significantly reduced by moxibustion or moxibustion combined with 1-MT treatment, which suggests that moxibustion may improve depressive behavior by inhibiting IDO1 protein production, mediating KP, and by modulating neuronal activation and microglia activation. Owing to the similar mechanisms of action of moxibustion and 1-MT, the combination of the two failed to increase the effect.

Previous studies (Ogyu et al., 2018) have revealed KP disorders in depressed patients with reduced levels of KYNA and KYN and elevated levels of QUIN in patients who were not taking antidepressants. These findings suggested the involvement of the KP pathway in the development of depression. The principal metabolite of KP, QUIN, is mainly produced in microglia (Guillemin et al., 2005), and is an agonist of *N*-methyl-D-aspartate (NMDA) receptors and a neuroexcitatory toxin found mainly in the forebrain (Schwarcz et al., 2012). Available evidence suggests that a series of neurotoxic effects caused by excessive activation of NMDA receptors in glutamate receptors is one of the important mechanisms of depression (Murrough et al., 2017). Among the NMDA receptor subtypes, the subtypes 2A and 2B show the highest affinity for QUIN and are widely distributed in the hippocampus. Moreover, quinolinate phosphoribosyl transferase (QPRT) activity is low in the hippocampus, and QUIN is difficult to metabolize in the hippocampus; therefore, the neurotoxicity of QUIN is most likely to affect the hippocampus (Lugo-Huitron et al., 2013). As a result, we speculate that the depressive behavior in CD model rats may be stimulated by dyshomeostasis of KP in hippocampal regions.

Studies have shown that the effect of acupuncture on depressive behaviors may be related to the regulation of the KP pathway. Kwon et al. (2012) induced an inflammatory depression model in mice with BCG vaccine and acupunctured the Sanyinjiao (SP6) and Shenmen (HT7) acupoints in mice. They found that acupuncture reduced depressive behaviors in the model mice and affected both serum KP and hippocampal dopamine. Li et al. (2019) found a reduction of depressive behavior in moxibustion rats in a similar model, and observed a strong correlation between hippocampal Kyn/Trp ratio and behavioral scores. Consistent with the above, the model group in our study showed a significantly increased KYN/TRP ratio and significantly decreased KYNA/KYN and KYNA/QUIN ratios compared to the control group; however, all of these metabolite ratios were reversed by moxibustion or co-treatment with 1-MT. Several studies have supported the association of KYN/TRP with CD disease activity, C-reactive protein, hematocrit (Gupta et al., 2012), and other important indices. Several studies have used KYNA/QUIN as a putative neuroprotective factor. Serum KYNA/QUIN was lower in depressed patients and showed a negative correlation with low pleasure, while a positive correlation was observed with hippocampal and amygdala volume in depressed patients (Savitz et al., 2015a,b). Therefore, our findings demonstrated that moxibustion may alleviate depressive behaviors in rats by modulating KP in the hippocampus. The mechanisms of action of moxibustion and 1-MT may be similar, so the combination of the two failed to increase the effect.

In summary, our findings suggested that moxibustion may be beneficial in improving inflammation and metabolic disorders in the hippocampal region by reducing excessive activation of hippocampal neurons and microglia, thereby inhibiting KP. The current study established a TNBS-induced model of CD and showed that depression in CD may be associated with neurotransmitter disturbances along the gut-brain axis. KP products can be modulated by moxibustion, suggesting that metabolic changes and behavior in the central nervous system may be influenced by moxibustion through KP along the gut-brain axis, which may result in improvement in depressive behavior. Our study provides a theoretical basis for early intervention for depressive behaviors, and contributes to the understanding of the mechanisms of depression and even other neuropsychiatric disorders.

However, some limitations of our study should be acknowledged. First, the pain induced by insertion of the needle for administration of each dose is liable to induce stress (Jin et al., 2019). Secondly, the immunofluorescence and Western blot assay used in the study were restricted to the analysis of protein expression, and RT-PCR was not applied to detect the corresponding gene expression levels. In addition, the effects of moxibustion intervention on NMDA receptors in microglia and neurotransmitter metabolism need to be further studied. Third, no IDO1 activator was applied in this study to further validate the effect of moxibustion. Fourth, gut microbes are also involved in one of the pathways of tryptophan metabolism (Agus et al., 2018), and future studies should investigate whether moxibustion affects IBD depression through additional pathways.

## Conclusion

In the present study, moxibustion at ST25, CV6, and GV20 acupoints alleviated CD depression-like behavior, which may be related to the inhibition of intestinal pro-inflammatory factors as well as IDO1 expression, which in turn inhibited excessive KP metabolism of tryptophan, thereby attenuating the effect of KP metabolites in the gut-brain axis and inhibiting hippocampal neuronal activation and microglia activation. The mechanism by which moxibustion alleviates depressive behavior in rats with TNBS-induced colitis may involve the bidirectional regulatory mechanism between the brain and the gut. Although moxibustion is not considered as a routine antidepressant therapy, our study provides evidence for the clinical application of moxibustion as an adjunctive treatment for CD-related depression.

## Data availability statement

All datasets generated for this study are included in the article/supplementary material.

## Ethics statement

This animal study was reviewed and approved by the Experimental Animal Ethics Committee of the Shanghai University of Traditional Chinese Medicine (No. PZSHUTCM200918016).

## Author contributions

CB conceptualized the study and designed the experiments. LC, DY, JH, and YM performed the experiments. HZ performed the data analysis. CB and JH drafted this manuscript. HW, HL, YS, and YL supervised the study and gave critical revision of the manuscript. All authors approved the final version of the manuscript.
